# Structural and interaction analysis of the Rrp5 C‐terminal region

**DOI:** 10.1002/2211-5463.12495

**Published:** 2018-08-30

**Authors:** Natacha Pérébaskine, Stéphane Thore, Sébastien Fribourg

**Affiliations:** ^1^ INSERM U1212 CNRS 5320 Université de Bordeaux France

**Keywords:** homopolymeric RNA, pre‐40S maturation, protein interaction, ribosome, ribosome polygenesis, RNA binding, Rrp5, TetratricoPeptide Repeat, TPR

## Abstract

Rrp5 is an essential factor during the ribosome biogenesis process. The protein contains a series of 12 S1 RNA‐binding domains followed by a TetratricoPeptide Repeat (TPR) domain. In the past, several studies aiming at defining the function of the TPR domain have used nonequivalent Rrp5 constructs, as these protein fragments include not only the TPR module, but also three or four S1 domains. We solved the structure of the Rrp5 TPR module and demonstrated *in vitro* that the TPR region alone does not bind RNA, while the three S1 domains preceding the TPR module can associate with homopolymeric RNA. Finally, we tested the association of our Rrp5 constructs with several proposed interactors, in support of cryo‐EM‐based models.

**Coordinates:**

Atomic coordinates and structure factors have been deposited to the Protein Data Bank under the accession number 5NLG.

**Abbreviation**
TPR: TetratricoPeptide Repeat

Ribosome biogenesis is a complex process starting in the nucleolus with the transcription by RNA polymerase I of a long preribosomal RNA (pre‐rRNA) containing the future 18S, 25S and 5.8S RNA species, while the fourth pre‐rRNA species (5S) is transcribed by the RNA polymerase III 1, 2. This process ends in the cytoplasm after a series of intertwined maturation events including methylation, pseudo‐uridylation, exo‐ and endonucleolytic cleavages at discrete sites, large conformational reorganization and transport through the nuclear pore complex. The different cleavages occur either co‐transcriptionally or post‐transcriptionally following the release of the 35S pre‐rRNA 3, 4, 5. The early steps of 18S, 28S and 5.8 pre‐rRNA maturation are common until the so‐called A2 cleavage, which segregates the 18S and 25S/5.8S rRNA biogenesis pathways.

Among the many trans‐acting factors that are strictly required for ribosome synthesis in yeast, Rrp5 is essential for the maturation of the two ribosomal subunits and, as such, has been found associated with early preribosomal particles 6. Rrp5 is a large multidomain protein that contains 12 repeats of the S1 RNA‐binding domain at its N terminus and 7 TetratricoPeptide Repeat (TPR) motifs at its C terminus. Genetic depletion of Rrp5 inhibits the early cleavages at sites A0, A1 and A2 during the 18S rRNA synthesis pathway and also at site A3 on the pathway of 5.8S/25S rRNA synthesis 7. In contrast, Rrp5 does not affect the endonucleolytic processing of B1L on the alternate pathway of 5.8S/25S processing, showing that the effects on A3 cleavage are specific. Rrp5 is encoded by an essential gene and carries functions that can be separated to solely its N‐terminal and C‐terminal domains as demonstrated through trans‐complementation experiments 7, 8, 9. This important role in coordinating 40S and 60S ribosomal subunit biogenesis has been successfully corroborated by the determination of 35S pre‐rRNA binding sites for the full‐length as well as the N‐ and the C‐terminal Rrp5 truncations 10. *In vivo*, the N terminus is required for A3 cleavage, while the C terminus is necessary for A0‐A2 cleavages. Consistently, the C‐terminal domain has been cross‐linked to sequences flanking the A2 cleavage site as well as to various snoRNA required for A2 cleavage such as U3, U14, snR30 and snR10 9.

Rrp5 has been proposed to interact with a number of trans‐acting factors 7, 9, 10, 11. This list includes direct interactions visualized using recombinant proteins such as the RNA helicases Has1 12 or Rok1 11, 13, as well as the Noc1–Noc2 complex 14. Additionally, some trans‐acting factors including the GTPase Bms1, various Utp subunits and ribosomal proteins have been linked to Rrp5 but most probably as indirect binders 15. Recently, it has been shown that Rrp5 dissociates from the pre‐40S subunit in a Rok1/ATP‐dependent fashion 11.

In keeping with its central role in ribosome biogenesis, the Rrp5 protein has been extensively studied. Hence, a number of studies have been performed using constructs corresponding to the 3 last C‐terminal S1 domains plus the TPR region 9, 10, 14, 16, but little reports have been done with separate S1 and TPR domains. Usually, S1 domains are prone to nucleic acid binding, while TPR domains are seen as protein–protein interaction modules. Here, we present the crystal structure of the sole Rrp5 TPR domain. The atomic details are consistent with the recently published structure by the Karbstein's laboratory 12 and from the cryo‐EM‐based model 17, 18.

On that basis, we have re‐examined the RNA‐binding capacity of the Rrp5 C‐terminal region by testing not only the TPR or the S1 domains but also their combination. Through these experiments, we delineate the S1 repeats as the region of Rrp5 responsible for RNA interaction, and we exclude the TPR domain as a contributor to single‐stranded RNA binding. Moreover, we have tested the capacity of the same Rrp5 C‐terminal regions to interact with several trans‐acting factors (Utp22, Has1, Kre33 and the RNA helicase Rok1), previously suspected or reported binders of the full‐length Rrp5 protein 10. We only managed to confirm the direct and weak interaction of Rrp5 with Rok1 in an ATP‐dependent manner. Altogether, the presented data confirm the atomic structure of the TPR domain of Rrp5 and disfavour RNA‐binding or protein‐interacting capacity for this region.

## Materials and methods

### Constructs

The *Saccharomyces cerevisiae* RRP5 gene was amplified from genomic DNA and inserted into the *Nde*I and *BamH*I sites of a modified pET (Novagen) plasmid 19 to produce an N‐terminal His‐tagged protein containing a TEV cleavage site or tag‐free recombinant proteins. All Rrp5 constructs (residue 1083–1729, residue 1083–1343 and residue 1400–1729 of the *S. cerevisiae* protein), as well as the Utp22, the Has1, the Kre33 and the Rok1 constructs, were sequenced to ensure the absence of mutations.

### Protein expression and purification

The various Rrp5, Utp22, Has1, Kre33 and Rok1 constructs were expressed and purified using the same protocol. Plasmids containing the indicated ORF were first transformed into *E. coli* Rosetta2 (DE3) cells. The cultures were grown at 37°C in TB medium supplemented with 100 mg·L^−1^ ampicillin and 33 mg·L^−1^ chloramphenicol. Cells were induced overnight at 15°C with 0.25 mm IPTG, then collected by centrifugation at 4500 ***g*** and resuspended in loading buffer (25 mm Tris pH 7.5, 150 mm NaCl). Cell pellets were lysed with an EmulsiFlex‐C3 (Avestin) and centrifuged at 50 000 × ***g*** for 45 min at 4°C. The clarified cell lysate was mixed with His‐Select Co^2+^ or Ni^+^‐NTA resin (Sigma) for 30 min at 4°C. The resin containing bound proteins was washed with 10 column volumes of loading buffer. At this point, beads were either used directly to perform pull‐down experiments or eluted with a 10 mm to 250 mm imidazole linear gradient.

Following the elution from the resin, the Rrp5 constructs were incubated with 1 mm DTT, 0.5 mm EDTA in the presence of tobacco etch virus (TEV) protease overnight at 16°C to cleave the tag. Samples were then diluted with loading buffer and re‐incubated with Ni^+^‐NTA resin to remove the TEV protease, cleaved tag and uncleaved proteins. The flow‐through was collected and ran onto a Superdex 200 gel filtration HR16/60 column (GE Healthcare, Paris, France) equilibrated in binding buffer (50 mm HEPES pH 7.0, 300 mm NaCl, 5% glycerol, 1 mm DTT). Protein peaks were analysed by SDS/PAGE, and fractions containing the proteins were pooled and concentrated to ~ 0.6–0.8 mg·mL^−1^.

The Rrp5 (residue 1400–1729) protein fragment used for crystallization was purified by a size exclusion chromatography over a Superdex 200 gel filtration HR16/60 column (GE Healthcare) equilibrated in 25 mm Tris pH 7.5, 150 mm NaCl and 1 mm DTT. The seleno‐methionine‐substituted Rrp5 protein fragment was purified as the native protein with the exception that the plasmid was initially transformed into the *E. coli* B834 cells and expression performed in minimal medium supplemented with seleno‐methionine at 50 mg·L^−1^.

### Crystallization and structure determination

The native and Se‐substituted protein samples were concentrated to 5 mg·mL^−1^ and crystallized in 16% to 26% PEG 1000, 0.1 m Na_2_HPO_4_/KH_2_PO_4_, pH 6 at 20°C using the sitting drop method. Crystals were transferred to a cryoprotectant containing 35% PEG 1000, flash‐frozen in liquid nitrogen and maintained at 100 K in a nitrogen cryo‐stream during data collection.

Crystals belong to the space group *P*3_1_21 with unit cell dimensions a=b = 114.71 Å, c = 68.01 Å and contain one molecule per asymmetric unit. The structure of Rrp5p (1400–1729) was solved by SAD using seleno‐methionine‐substituted protein crystals. Datasets were reduced using XDS 20. Four selenium sites were located with SHELXCD suite 21, and their positions were refined with Phaser 22. The initial model was automatically built using Bucanneer 23, refined with BUSTER 2.10 24 and manually adjusted with Coot 25. The final model has good stereochemistry (Table 1) and corresponds to amino acid 1408 to 1418 and 1456 to 1721.

**Table 1:** Crystallographic data and refinement statistics. Numbers in brackets refer to the highest resolution shell

	Rrp5 (1400–1729) Se‐Met	Rrp5 (1400–1729)
Data collection statistics		
Beamline	Proxima 1	Proxima 1
Space group	*P*3_2_21	*P*3_2_21
Unit cell parameters		
a, b, c (Å)	114.71, 114.71, 68.01	114.01, 114.01, 65.74
α, β, γ (°)	90, 90, 120	90, 90, 120
Wavelength (Å)	0.9789	0.9801
Resolution range (Å)	50 ‐ 3.2 (3.42 – 3.20)	39.48 – 2.35 (2.41 – 2.35)
Total reflections	98 614 (17 555)	106 545 (6629)
vUnique reflections	8765 (1569)	20 756 (1491)
Rpim (%)	4.3 (31.2)	10.5 (94.7)
I/σI	11.0 (11.2)	13.17 (1.72)
Completeness (%)	100.0 (100.0)	99.8 (98.6)
Redundancy	11.3 (11.2)	5.1 (4.4)
Overall B factor (Å^2^)		40.8
Mosaicity	0.55	0.96
CC 1/2	100 (85.0)	0.998 (0.664)
Se sites	4	–
FOM acentric (before solvent flattening)	0.353/0.632	–
Refinement statistics		
Rfree (%)		24.08
Rwork (%)		19.78
No. of non‐H atoms		2420
Protein		2269
Water		151
Average B factor (Å^2^)		52.01
Ramachandran (%)		
Preferred regions		97.07
Allowed regions		2.56
Outliers		0.37
Rmsd bond lengths (Å)		0.009
Rmsd bond angle (°)		0.89
PDB		5NLG

### *In vitro* Poly(U) or Poly(C) RNA binding assays

Polyuridylic acid–agarose (poly‐U, Sigma ref. P8563) or polycytidine–agarose (poly‐C, Sigma ref. P9827) beads were used for the assay. The beads were equilibrated 5 times in 500 μL of reaction buffer (50 mm Tris pH 8, 150 mm NaCl, 5 mm MgCl_2_ and 0.1 mg·mL^−1^ BSA). Then, 100 μL of protein at a concentration of ~ 1 mg·mL^−1^ in the reaction buffer was added to 100 μL of equilibrated beads and incubated on ice for 30 min. Unbound proteins were removed by washing the beads five times with wash buffer (50 mm Tris pH 8, 150 mm NaCl, 5 mm MgCl_2_). The bound fraction was eluted by addition of 20 μL of 3 × Laemmli buffer to 50 μL of washed beads. The samples were then analysed by electrophoresis on 12.5% SDS/PAGE.

### Pull‐down assays

Approximately 2–4 μg of the indicated protein‐bound beads (or clean beads) was washed 3 times with wash buffer and mixed with 2–4 μg of its putative partners, that is the Rrp5 constructs at 0.6–0.8 mg·mL^−1^ concentration, in a final volume of ~ 100 μL. After 30 min of incubation in the ice, the beads were washed 4 times with 900 μL of wash buffer. About 30–50 μL of wash buffer was left in the tube and 15–20 μL of SDS/PAGE loading dye was directly added. Input and bound fractions from the pull‐down experiments were analysed on a 12.5% SDS/PAGE gel. Aliquots of protein‐bound beads (10 μL) or protein used for the pull‐down experiments (10 μL at 0.6–0.8 mg·mL^−1^) were loaded on a separate 12.5% SDS/PAGE gel shown in the Fig. S1.

## Results

### Overall structure of Rrp5 TPR domain

**Figure 1:**
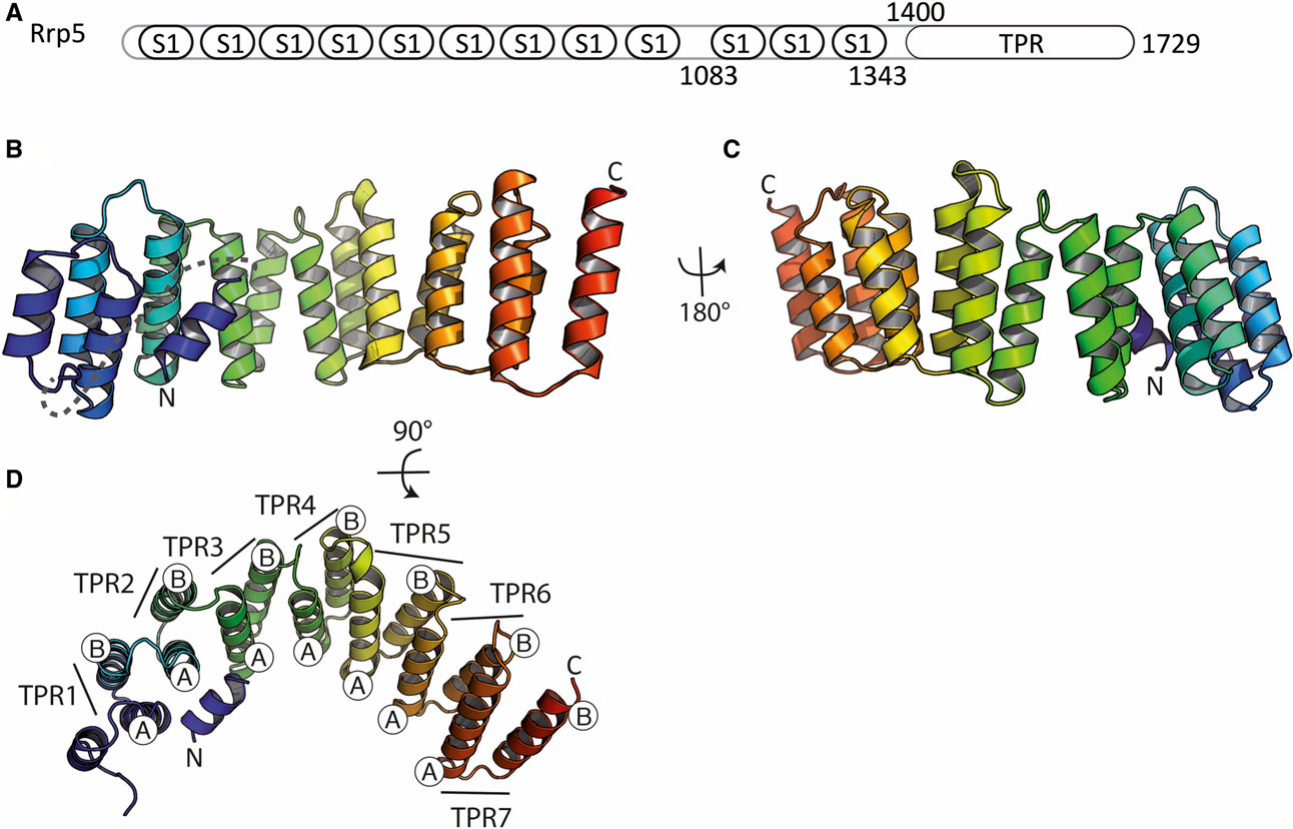
Overall structure of Rrp5 TPR domain. (A) Schematic view of the various domains present in the Rrp5 protein. (B) and (C) Side views of the X‐ray structure showing the TPR repeats present in the Rrp5 polypeptide. The helices are coloured from the N terminus in blue to the C terminus in red. The disordered loop between residues 1419–1456 is indicated as a grey dotted line, and the protein termini are labelled by N and C, respectively. (D) Top view of the TPR rod with individual TPR‐repeat A and B α‐helices numbered and indicated. The atomic structure is shown using the cartoon representation. All structural panels were prepared with the program Pymol 33.

We started the expression and purification of various Rrp5 domains encompassing the TPR with or without the last three C‐terminal S1 domains as used in previous analysis 10, 11, 16. The TPR‐repeat containing region (residue 1400–1729) was produced and purified as described in the Materials and Method. The purified protein was crystallized, and X‐ray diffraction data were collected up to 2.35 Å resolution. The structure was solved by a SAD phasing experiment on a Se‐Met‐substituted protein crystal. The final model was refined to a Rfree of 24.08% (Table 1). It comprises 276 residues of 329 from the construct and spans from residue 1408 to 1418, forming a short α‐helix, connected by an unresolved linker to the region 1456–1721. The last eight C‐terminal residues could not be modelled in the density (Fig. 1 and Fig. S2). Overall, the structure is composed of 14 antiparallel α‐helices forming a seven TPR‐repeat domain plus the additional α‐helix located at the concave surface of the TPR domain and interacting with TPR 1, 2 and 3 (Fig. 1). This structure is similar to the recently published ones with an overall r.m.s.d. of 0.375 Å and 0.344 Å over 276 and 272 Cα carbon, respectively (PDB codes 5C9S and 5WWM) 12, 18.

### Surface properties of Rrp5 TPR domain

**Figure 2:**
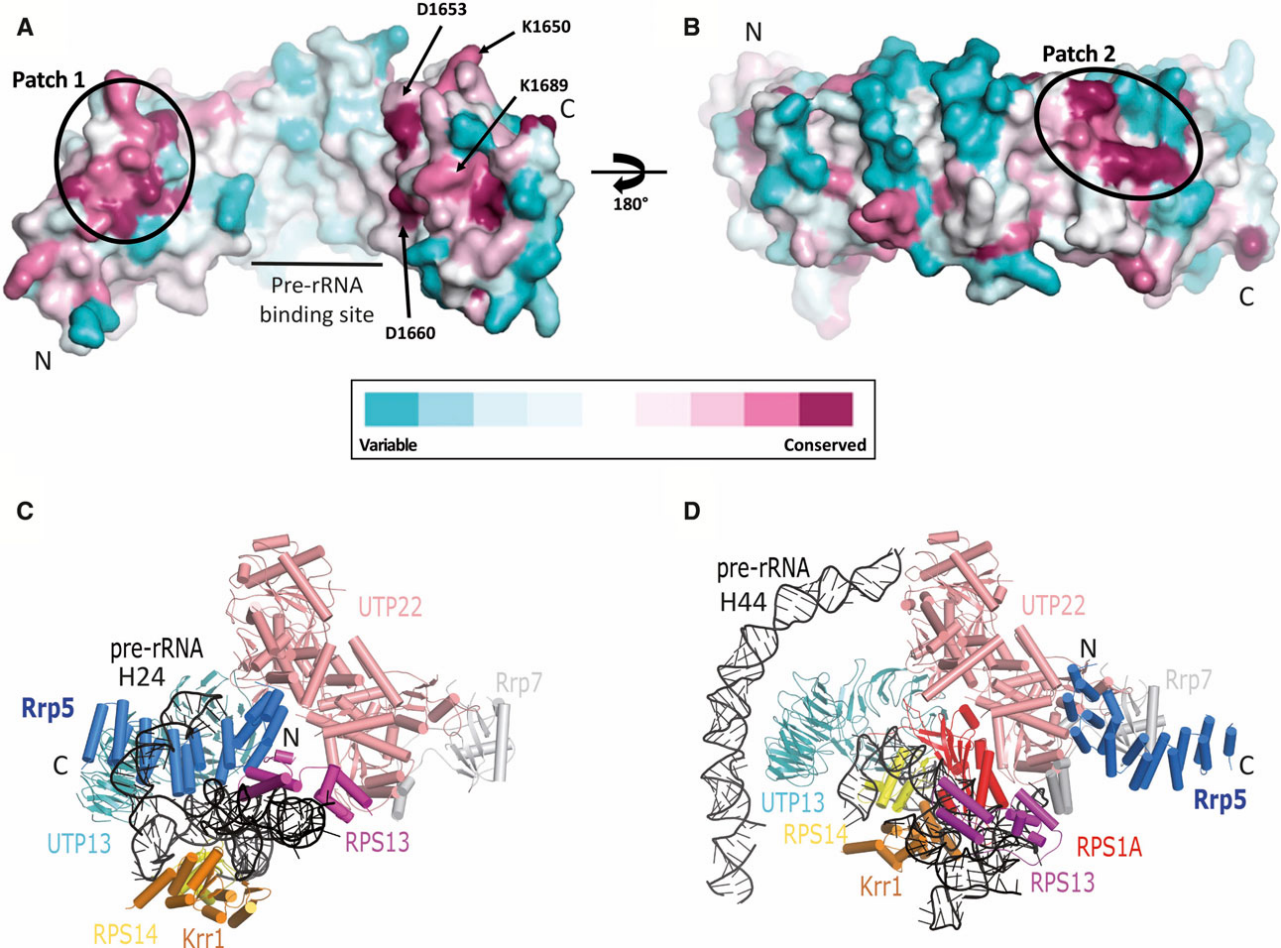
Surface conservation of Rrp5 residues and its location in the preribosomal particles. (A) Surface representation of the residue conservation in the Rrp5 protein family. The surface is coloured from blue to red (variable to conserved) and represented under the same orientation as in Fig. 1. (B) Same as in the panel A but rotated by 180°. The calculation has been performed with the ConSurf server 34 using the Rrp5 TPR sequence as reference to find 150 sequences sampled through homologous organisms with overall identity ranging from 35 to 95%. Sequence alignment is shown in Fig. S2. Patches 1 and 2, pre‐rRNA binding site and conserved residues discussed in the text are labelled as well as the N‐ and C termini. (C) and (D) Cartoon representations of pre‐40S and pre‐90S particles as reported by Barandun *et al*. (PDB code 5WLC) 35 and Sun *et al*. (PDB code 5WYJ), respectively 18. The two models were superimposed using Utp22 as a reference. Only the direct neighbourhood of Rrp5 is displayed. Rrp5 N termini and C termini are indicated.

Rrp5 is known to be involved in a variety of interactions within the ribosome biogenesis pathway including protein and RNA binding partners 10, 11, 12, 18. Given that Rrp5 is conserved from yeast to mammals, it is likely that conserved surface areas correspond to the functional regions. To possibly identify key determinants of Rrp5 function, we plotted the invariant residues at the surface of the Rrp5 TPR domain structure using the ConSurf server 26. Several patches of conserved residues were identified and located at the N terminus and C terminus of the TPR domain on the concave and the convex faces (Fig. 2). At the N terminus, residues N1471 to S1475 and the loop between R1508 to E1512 form a conserved and continuous surface on the concave face of the TPR rod (indicated as patch 1 on Fig. 2A). At the other end, residues E1635, E1642 and R1677 form another hotspot of conservation on the convex face spreading over TPR motif 5 and 6 (labelled patch 2 on Fig. 2B). Additional conserved residues are found on both sides of the TPR rod and include residues K1650, D1653, D1660 and K1689 (Fig. 2A). Recent cryo‐EM reconstructions have located the Rrp5 TPR module next to the Utp22/Rrp7 protein complex 17, 18 (Fig. 2C,D). The interaction between the Rrp5 and the Utp22 protein would involve the conserved residues identified on the convex face (Fig. 2A,C Fig. S2). Besides protein–protein interaction, cryo‐EM‐based model also suggests that the pre‐rRNA helix 24 binds to the Rrp5 TPR module using the above‐mentioned conserved residues on the Rrp5 TPR rod (Fig. 2C,D) 24. However, this site is partially blocked by a short helix in our crystal structure (Fig. 1). The peptide had to be removed from the cryo‐EM‐fitted atomic model 12, 17. This suggests that Rrp5 has to adapt its overall conformation concomitantly with its binding to the pre‐40S particle, as for example with Rio2 binding to RNA 27.

### *In vitro* RNA binding properties of Rrp5 TPR domain

**Figure 3:**
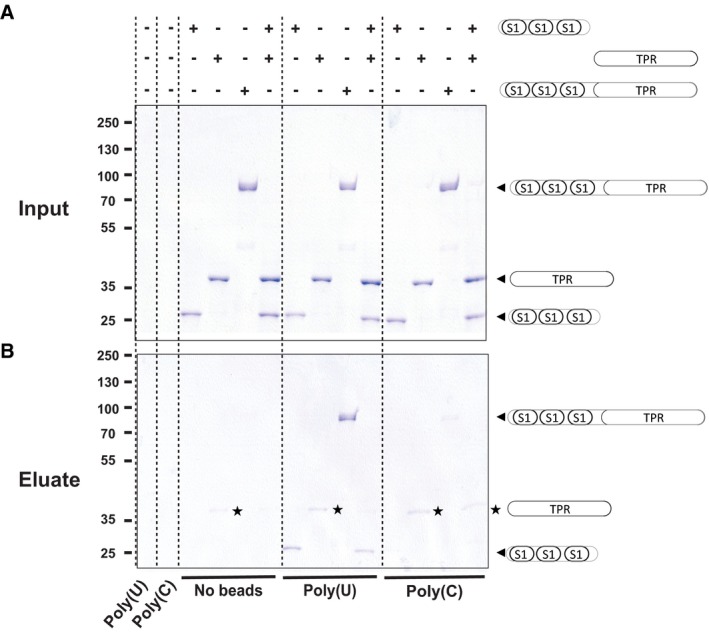
RNA pull‐down experiments with Rrp5 C‐terminal constructs. RNA pull‐down experiments have been performed by incubating poly(U) or poly(C) Sepharose resin with various purified Rrp5 C‐terminal constructs as described in the Material and Method section. The top panel represents the input sample, while the bottom panel represents the bound fractions. The Rrp5 constructs are shown as cartoons.

Rrp5 has been reported to interact simultaneously with the 5′‐sequences flanking the A3 cleavage site and to the 3′‐sequences flanking the A2 cleavage site of the pre‐35S rRNA 10, 16. Rrp5 TPR domain has also been shown to interact with the pre‐rRNA helix 24 as indicated above 17. We performed a pull‐down assay using poly(U) or poly(C) RNA Sepharose beads and purified Rrp5 fragments to assess whether the TPR domain is sufficient to stably bind homopolymeric single‐stranded RNA *in vitro* 28. Rrp5 fragment (1083–1729) comprising the 3 last S1 domains and the TPR region, Rrp5 (1083–1343), which contains only the last 3 S1 domains, and Rrp5 (1400–1729) corresponding to the TPR region alone were incubated with the RNA‐bound beads (Fig. 3A). After 30 min of incubation on ice, the beads were extensively washed and bound proteins were analysed by SDS/PAGE and revealed by Coomassie Blue staining (Fig. 3B). As expected, the TPR domain was not sufficient to observe a stable complex with RNA although limited nonspecific binding to the plastic tubes was observed. Poly(U) association was detected only with the construct containing the three S1 domains, while poly(C) resin did not bind any of the constructs, demonstrating some specificity of interaction with poly(U) sequence (Fig. 3B). With the recombinant Rrp5 fragments, we could confirm that poly(U) association is mediated by the last three S1 domains with apparently no significant contribution by the TPR region 28. This observation is in agreement with previous reports where removal of the three last S1 domains was shown to weaken the interaction of Rrp5 with the pre‐40S particle *in vivo,* and with 40S subunit *in vitro* 12.

### *In vitro* protein–protein interactions

**Figure 4:**
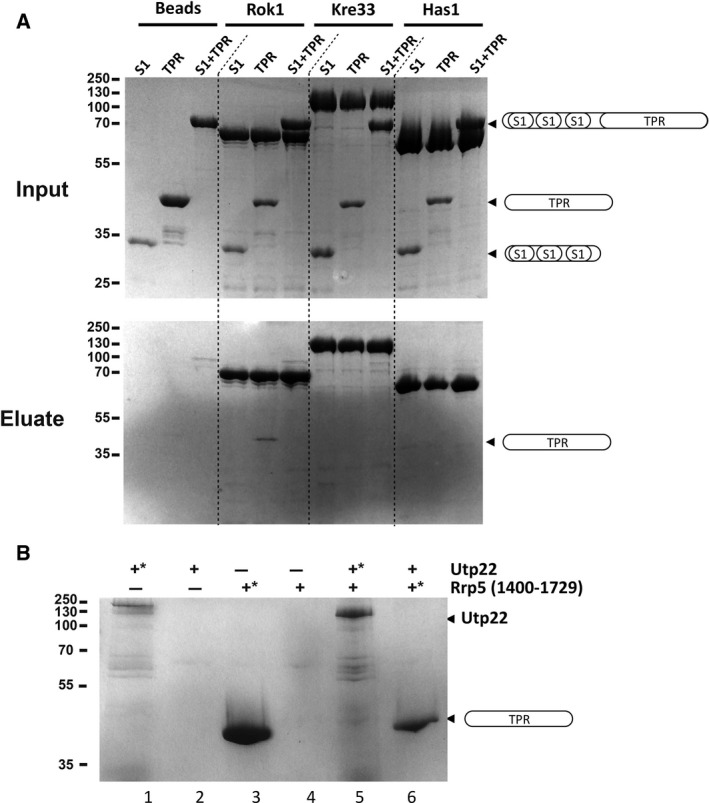
*In vitro* pull‐down assays between Rrp5 C‐terminal constructs and specific trans‐acting factors involved in rRNA biogenesis. (A) Three trans‐acting factors Rok1, Kre33 and Has1 were overexpressed in bacteria, bound on Ni^+^‐NTA beads and analysed on a 12.5% SDS/PAGE visualized by Coomassie Blue staining. After extensive washes, aliquots of the protein‐bound beads were incubated with the different Rrp5 C‐terminal constructs as described in the Material and Method section. The top panel shows the input samples and the bottom panel the eluate fractions. The Rrp5 constructs are displayed as cartoons as in Fig. 1A. Incubation with Rok1 was performed in the presence of 2 mm ATP. (B) SDS/PAGE analysis of Utp22 and Rrp5 TPR domain association by co‐expression. His‐tagged or untagged versions of the Utp22 protein (lane 1, 3, 5 and 6) or the Rrp5 TPR domain (lane 2, 4, 5 and 6) were expressed alone and together in *E. coli*. Lysates were run on Ni^+^‐NTA beads and eluates analysed by SDS/PAGE. An asterisk ‘*' indicates that the (co‐) expressed protein was His‐tagged.

Rrp5 is known to interact with a number of protein maturation factors from both the pre‐40S and the pre‐60S subunits including Utp22, Rok1 or Has1 10, 12. To test whether the TPR domain of Rrp5 is responsible for a direct interaction with these proteins, we performed *in vitro* pull‐down experiments (Fig. 4). Full‐length Rok1, Kre33, Has1 and Utp22 were first expressed in *E. coli* Rosetta2 cells as His‐tagged proteins. The proteins were incubated with Nickel‐affinity resin and protein‐bound beads analysed on a SDS/PAGE gel. Little background contamination by endogenous *E. coli* proteins was observed (Fig. S1). Then, the three Rrp5 constructs (Rrp5 1083–1729, Rrp5 1083–1343 and Rrp5 1400–1729) were incubated with the resin‐immobilized proteins. After extensive washing, the beads were analysed on a SDS/PAGE, but we could not visualized any additional bands corresponding to either of the Rrp5 constructs (Fig. 4A). Noteworthy, incubation with the Rok1 protein was performed in the presence of 2 mm ATP as it was recently suggested that Rok1 ATPase activity may be relevant for the stability of the Rrp5–Rok1 complex 12. Our incubation resulted in a very weak and not stoichiometric interaction between Rok1 and the Rrp5 TPR construct, which was not confirmed with the longer construct containing the S1 domains (Fig. 4A). Utp22 or Has1 were further co‐expressed with the Rrp5 TPR rod to verify whether the association may be dependent on the proteins being produced simultaneously, but we still did not observe any co‐purification (Fig. 4B and data not shown, respectively).

## Discussion

Rrp5 is a 193 kDa protein composed of 13 predicted modules (12 S1 domains plus one rod of 7 TPR motifs), which likely adopts an extended conformation *in vivo*. The capacity to rescue the rrp5 deletion phenotype using two nonoverlapping fragments containing either the N‐terminal or the C‐terminal region of the protein clearly argues for a modular organization, with the possible consequence of one domain to one function 9, 16. Many partners of Rrp5 have been identified in the past years including trans‐acting protein factors and pre‐rRNA fragments. Altogether, this list comprises Rok1, Noc1–Noc2 and Has1 as direct binders 11, 12, 14, and Utp10, Utp20, Utp21, Kre33, Rrp36 and Nop58 as potentially direct/indirect partners of Rrp5 9. However, their precise binding sites onto Rrp5 have only recently been determined 12. Experimentally measured electron density maps based on cryo‐EM preparation of pre‐40S particles allowed the positioning of the Rrp5 TPR domain in close proximity to the Utp22/Rrp7 complex 17. Moreover, helix 24 of the pre‐rRNA was fit well into a large fragment of electron density next to the concave surface of the Rrp5 TPR module 17. While Rrp7/Rrp5 complex existence was previously suggested 29, the pre‐rRNA helix 24 had never been found to be a partner or a binding site of Rrp5. Rather, the ITS1 sequence fragment containing the A2 and A3 cleavage sites was shown to bind to the S1 domains of Rrp5 preceding the TPR region 9, 10. Moreover, the Rrp5 fragments used in the previously mentioned rescue studies did not share the same boundaries, highlighting a potential discrepancy in the conclusions. Specifically, these boundaries between the N‐ and the C‐terminal fragments differ by 50 residues before the three S1 domains preceding the TPR module 10, 16.

To gain further insights into this putative network of interactions, we explored the structure, RNA binding and potential partner association of several Rrp5 C‐terminal constructs, using a conservative N‐terminal start with regard to the position of the last three S1 domains and of the TPR region 9. We determined the crystal structure of Rrp5 TPR domain using a construct from residues 1400 to 1729. We also used larger Rrp5 protein constructs, including the three S1 domains preceding the TPR rod, and did obtain crystals. However, due to the absence of electron density signal, the S1 motifs preceding the TPR motifs are invisible in our crystal lattices (data not shown). Our solved structure is composed of a N‐terminal short helix bound to the concave surface of a TPR module containing 7 individual motifs. Globally, our structure is similar to the previously published models 12, 18.

Residue conservation showed an important patch of co‐evolving amino acids covering the surface of TPR 1‐3 (Fig. 2). Interestingly, the same Rrp5 TPR 1‐3 region interacts with the Utp22/Rrp7 protein complex in a recent cryo‐EM structure 18. We tried to reproduce this interaction in an *in vitro* pairwise assay, but in our hands, Utp22 does not stably bind to the Rrp5 TPR fragment nor to the one including the three S1 domains (Fig. 4). This interaction might be too weak to be detected under our experimental conditions or may require physiological neighbouring factors such as Rrp7 or the ribosomal RNA. In an attempt to include Rrp7 in the *in vitro* assay, we tried although unsuccessfully to obtain Rrp7 from a recombinant source. Rrp7 production was poor with limited expression and stability (data not shown).

Several other proteins are known partners of Rrp5 but details of their interaction have so far eluded characterization, with the recent exception of the Rok1 protein 12. We also tried to reproduce these published interactions *in vitro* using our Rrp5 truncations 12. Unfortunately, none of our pull‐down assays or co‐expression experiments showed the formation of a stable and stoichiometric complex. These experiments therefore may be indicative that the S1 domains found outside our tested fragments are responsible for these interactions. In a final attempt to explore this hypothesis, we failed to purify/express full‐length Rrp5 or any constructs containing the first nine S1 domains, preventing us from directly testing the mentioned suggestion. Furthermore, we cannot rule out that these protein/protein interactions are too weak to be observed outside the physiological environment represented by the preribosomal particle, a situation reminiscent to the one found with the spliceosomal particles 30.

With respect to interaction with RNA, we and several groups failed to show any RNA‐binding capacity for the TPR motifs despite the cryo‐EM‐based observation of the helix 24 pre‐rRNA bound over the TPR motifs 2–4 17, 28. In fact, our RNA binding experiments clearly show that the three S1 domains rather than the TPR module are responsible for RNA association (Fig. 3). It is noteworthy to mention that despite the clear role of the S1 domains for RNA association *in vitro* 28, their removal abolishes pre‐rRNA processing at site A2 31.

The recent revolution in the cryo‐EM data collection leading to a much improved resolution allows tackling particles and complexes that are reluctant to crystallize and thought to be too labile, even in the recent past, to obtain a near‐atomic description. High‐resolution structures are still required to confidently explain the medium‐ to low‐resolution experimental electron density cryo‐EM maps. External controls besides a correlation coefficient value describing the model fit into the cryo‐EM density map should be used to confirm the large number of assignments proposed in recently published cryo‐EM reconstructions 23, 24, 26. In the present article, we did not manage to provide such an external validation for the Rrp5–Utp22 interaction.

Of course, *in vitro*‐based validation faces technical issues linked to a minimalistic strategy and weaknesses of the interactions due to incomplete reconstitution strategy or else, but its successful implementation is of interest to strengthen the confidence in the proposed models. This is especially true when the resolution does not allow for confident assignment of amino acid placement, or for peripheral regions from large RNP complexes with data of lower resolution. Our recommendation is nicely substantiated by a recently published article regarding the function of two trans‐acting factors, Krr1 and Dim2 32. Their identical fold and localization on the pre‐rRNA but their temporal binding during ribosome biogenesis required *in vitro* experimentation to validate their identification in the cryo‐EM models.

## Author contributions

SF and ST cloned the protein constructs. NP and ST purified and crystallized the protein. SF collected, treated the data, solved the structure, and build and refined the initial model. ST refined the structure and performed RNA pull‐down experiment. SF and ST performed the protein pull‐down experiments. SF supervised the work. SF and ST wrote the manuscript. All authors corrected the manuscript.

## Supporting information


**Fig. S1.** SDS/PAGE analysis of the proteins used for the pull‐down experiments shown in Fig. 4. Lanes 2, 3 and 4 contain Ni^+^‐NTA beads loaded with the tested proteins before the incubation with the Rrp5 constructs. Lanes 6, 7 and 8 show the purified Rrp5 constructs used for the pull‐down assays.

**Fig. S2.** Sequence alignment generated by the webserver Consurf. The alignment uses 150 homologous protein sequences with identity ranging from 35 to 95 per cent.
