# Supplementary figures and images for: Structural and interaction analysis of the Rrp5 C‐terminal region
Source: FEBS Open Bio. 2018 Aug 30;8(10):1605–14. doi: 10.1002/2211-5463.12495 (PMC6168700; doi:10.1002/2211-5463.12495)

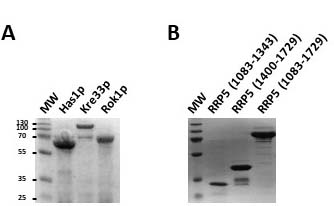

Supplement: Supplementary file 1 — Fig. S1. SDS/PAGE analysis of the proteins used for the pull‐down experiments shown in Fig. 4. Lanes 2, 3 and 4 contain Ni+‐NTA beads loaded with the tested proteins before the incubation with the Rrp5 constructs. Lanes 6, 7 and 8 show the purified Rrp5 constructs used for the pull‐down assays. [file FEB4-8-1605-s001.jpg]

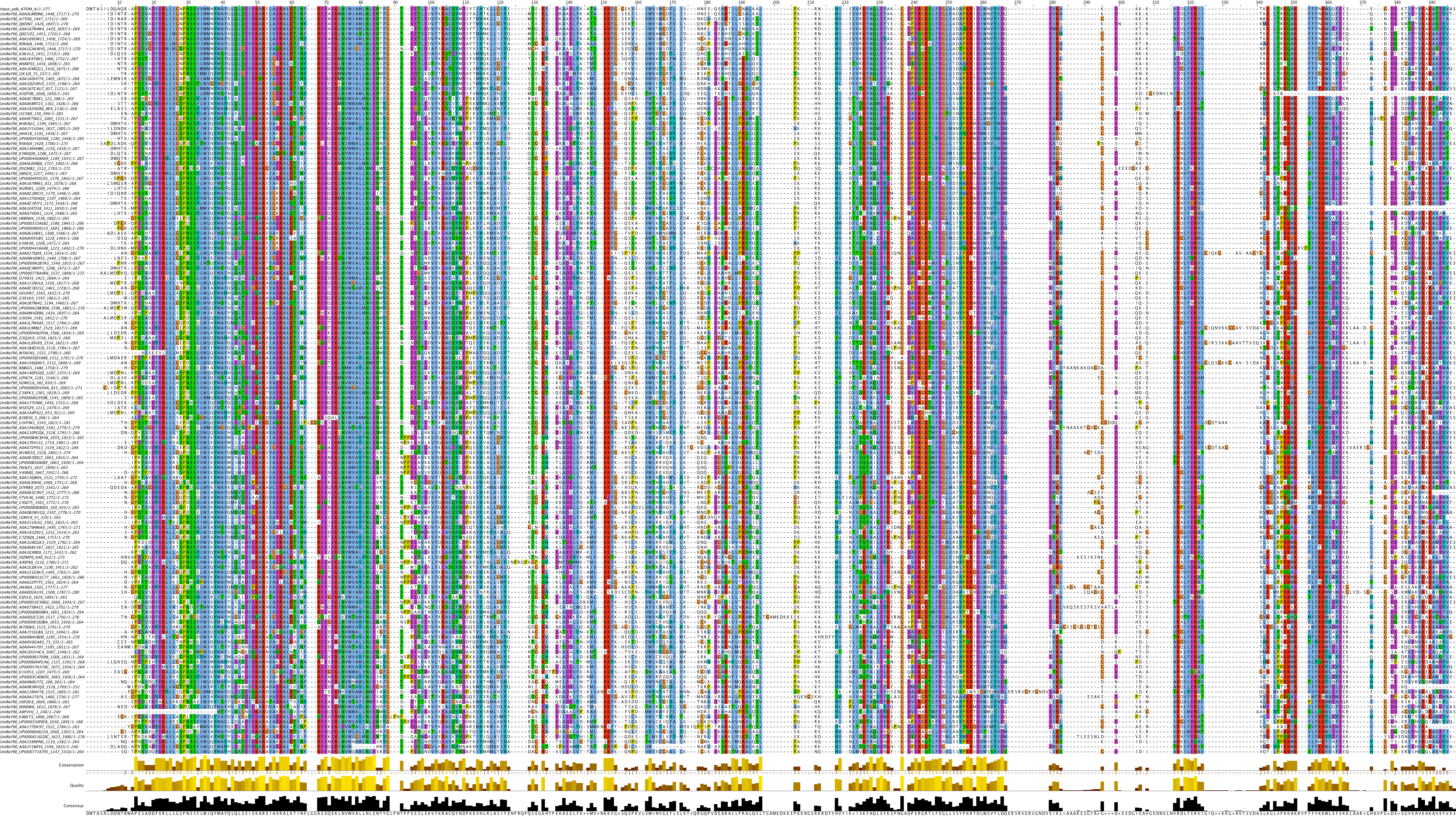

Supplement: Supplementary file 2 — Fig. S2. Sequence alignment generated by the webserver Consurf. The alignment uses 150 homologous protein sequences with identity ranging from 35 to 95 per cent. [file FEB4-8-1605-s002.png]
